# Human-Edited Generative AI-Assisted Multiple-Choice Questions in Postgraduate Family Medicine: Blinded Cross-Sectional Comparative Psychometric Study

**DOI:** 10.2196/100179

**Published:** 2026-08-10

**Authors:** Kai Ping Sze, Jia Qing Lim, Yng Miin Loke, Teck Yong Gabriel Ding, Wei Chieh Wee, Tongyuan Chen, Jingkai Lawrence Wu, Vivek Bansal, Qi Wei Fong, Minyang Chow

**Affiliations:** 1 LKC School of Medicine, Nanyang Technological University, Singapore, Singapore; 2 NHG Polyclinics, Population Health Campus, National Healthcare Group, 690 Yishun Ring Road, Singapore, 769567, Singapore, 65 63553000; 3 Clinical Education, National Healthcare Group, Singapore, Singapore; 4 Professional Training and Standards Division, Ministry of Health, Singapore, Singapore, Singapore

**Keywords:** generative artificial intelligence, ChatGPT, multiple-choice questions, medical education, family medicine, assessment, psychometrics, item analysis, distractor analysis

## Abstract

**Background:**

Generative artificial intelligence (GenAI) is increasingly used to draft multiple-choice questions (MCQs) for health professions education, but much evidence concerns raw model outputs, expert ratings, or item difficulty alone. Educators edit GenAI drafts before use, and whether such items are psychometrically ready for postgraduate assessment remains unclear.

**Objective:**

This study aimed to compare human-edited GenAI-assisted and educator-crafted MCQs for postgraduate Family Medicine Applied Knowledge Test–level assessment, examining difficulty, discrimination, reliability, distractor functioning, and participant perceptions.

**Methods:**

We conducted a blinded cross-sectional, within-participant comparative psychometric evaluation in Singapore. Sixty best-of-five single-best-answer MCQs were evaluated, 30 human-edited GenAI-assisted items and 30 educator-crafted items, topic-matched across postgraduate FM domains and randomized across 2 assessment sets. Eligible participants were postgraduate doctors enrolled in FM residency or postgraduate family medicine programs, preparing for the Applied Knowledge Test, and blinded to item origin; incomplete paired responses were excluded. Outcomes included paired total scores, score correlation and agreement, Kuder-Richardson Formula 20 reliability, item difficulty index, corrected point-biserial discrimination, distractor functioning, and perceived difficulty, clarity, and relevance. Analyses used paired-sample tests, Pearson correlation, Fisher exact tests, and item-level psychometric statistics, with α=.05 and Bonferroni correction within comparison families.

**Results:**

Of 74 participants, 73 completed both item sets and were included in the analysis. The final sample comprised 36 graduate diploma in FM trainees, 5 MMed FM trainees, and 32 FM residents. Paired-sample testing showed lower scores on GenAI-assisted than educator-crafted items (mean 19.12, SD 2.83 vs mean 21.10, SD 3.42 out of 30; mean difference −1.97, 95% CI −2.72 to −1.23; *P*<.001; Cohen *d*=0.62), indicating that GenAI-assisted items were not easier. Scores were positively correlated (*r*=0.49, 95% CI 0.30‐0.64; *P*<.001), but Bland-Altman analysis indicated limited agreement. Kuder-Richardson Formula 20 reliability was lower for GenAI-assisted items (0.38 vs 0.60). Mean difficulty index did not differ significantly (0.64 vs 0.70; mean difference −0.07, 95% CI −0.19 to 0.06; *P*=.29), and more GenAI-assisted items fell within the acceptable difficulty range (18/30, 60.0% vs 13/30, 43.3%). However, mean corrected point-biserial discrimination was lower for GenAI-assisted items (0.09 vs 0.18; mean difference −0.08, 95% CI −0.16 to −0.01; *P*=.04), and negative discrimination was more common (6/30, 20% vs 3/30, 10%). GenAI-assisted items also had more nonfunctioning and negatively discriminating distractors, although these differences were not statistically significant. Participant ratings of perceived difficulty, clarity, and practice relevance did not differ by origin.

**Conclusions:**

Human-edited GenAI-assisted MCQs can achieve plausible difficulty, but difficulty and surface acceptability did not ensure assessment readiness. Using trainee response data, this study extends work on raw outputs or expert opinion. GenAI should be used as a drafting adjunct within educator-led workflows prioritizing key verification, distractor engineering, pilot testing, empirical item analysis, and repair before item-bank or summative use.

## Introduction

### The Challenge of MCQ Development in Postgraduate Assessment

High-quality multiple-choice questions (MCQs) remain a cornerstone of written assessment in medical education. When well-constructed, single-best-answer MCQs can sample the curriculum broadly, be administered efficiently to large cohorts, and generate data suitable for psychometric analysis, thereby supporting meaningful score interpretation. However, defensible MCQ development is resource-intensive. It requires clinical expertise, assessment literacy, blueprint alignment, careful phrasing, plausible distractor construction, and iterative review. Programs therefore frequently struggle to scale item writing while maintaining item quality, especially where curricula evolve rapidly and assessment banks require regular renewal.

Postgraduate family medicine (FM) magnifies this challenge because assessments must sample acute, chronic, preventive, pediatric, geriatric, and palliative care while testing guideline-concordant management and applied judgment in undifferentiated primary care. In Singapore, the Family Medicine Applied Knowledge Test (FM AKT) is a national-level postgraduate written assessment comprising 180 single-best-answer MCQs across 2 papers [1].

In this setting, item quality affects more than content coverage. Ambiguous wording, unclear constructs, contestable keys, or poorly functioning distractors can distort score interpretation, reduce reliability, and undermine fairness, especially where scores may inform progression, remediation, or readiness for further training.

### GenAI-Assisted MCQ Development and Emerging Concerns

Generative artificial intelligence (GenAI) has attracted increasing interest as a potential solution to the item-writing burden. Large language models can rapidly generate clinical stems, answer options, explanations, and item variants, offering a scalable way to support educators in developing practice questions or refreshing item banks. Early empirical studies suggest that GenAI-generated MCQs can, in some settings, appear clinically plausible, receive favorable expert ratings, and achieve psychometric performance broadly comparable with human-authored items, particularly when outputs undergo expert review for factual accuracy, relevance, and alignment with learning objectives [2-7].

However, the emerging literature remains mixed. Recent syntheses have highlighted substantial heterogeneity in prompting methods, model versions, evaluation criteria, and the types of validity evidence reported [8,9]. Some studies have raised concerns that GenAI-generated MCQs may be easier than educator-authored items or more likely to assess lower-order cognitive processes, while others suggest more comparable performance after careful prompting and expert review [2-7]. This creates an important tension for assessment leaders: GenAI may accelerate item production, but it remains unclear whether the resulting items are sufficiently robust for assessment use.

A further concern is that GenAI-generated MCQs may contain subtle vulnerabilities that are not obvious during surface review. GenAI can produce fluent stems and plausible-sounding answer options, but fluency is not the same as assessment quality. Effective MCQs require more than a correct key and grammatically acceptable options. They require distractors that represent authentic misconceptions, realistic clinical reasoning errors, or plausible near-miss decisions. If distractors are too implausible, they may become nonfunctioning. If they are ambiguous or conceptually misaligned, they may attract higher-performing candidates and reduce item discrimination. These risks are particularly relevant because recent work has described format-specific pitfalls in using MCQs to evaluate GenAI-generated educational content, including cueing, overestimation of reasoning, and “hallucination-adjacent” distractors [10].

### From Raw AI Output to Human-Edited Assessment Items

A central practice-relevant gap is that most real-world implementations do not use raw GenAI outputs directly. Instead, educators typically use GenAI to generate draft items and then edit those items for clinical correctness, local guideline concordance, phrasing, relevance, and alignment with intended learning outcomes. The practical question is therefore not simply whether GenAI can generate MCQs. It is whether human-edited GenAI-assisted MCQs function equivalently to educator-crafted items when answered by the intended learner population.

This distinction matters because routine human editing may correct visible problems without resolving deeper psychometric vulnerabilities. An educator may be able to improve factual accuracy, remove obvious wording flaws, and ensure that the keyed answer is clinically defensible. However, these steps do not necessarily guarantee that the item discriminates between stronger and weaker candidates, that all distractors function as intended, or that the item contributes positively to internal consistency reliability. In other words, an item may be difficult enough and appear relevant to trainees, yet still be unsuitable for item-bank use if its option set does not support defensible score interpretation.

This distinction also reframes the concern that GenAI-generated MCQs may be “too easy” or too low-order. Difficulty is an important property, but it is not synonymous with assessment readiness. A recall-based item can be difficult if it tests obscure knowledge, while an applied clinical item can be easy if the decision is obvious. Conversely, a difficult item may still perform poorly if the construct is unclear, the key is contestable, or distractors create ambiguity. For GenAI-assisted MCQ development, the critical question is therefore 2-fold: whether edited GenAI-assisted items can achieve appropriate postgraduate difficulty, and whether items that achieve such difficulty also demonstrate adequate discrimination and distractor functioning.

### Study Aim and Contribution

In this study, we conducted a blinded psychometric comparison of human-edited GenAI-assisted and educator-crafted MCQs targeted at the postgraduate FM AKT level. Using trainee response data, we compared participant total scores, internal consistency reliability, item difficulty, corrected point-biserial discrimination, distractor functioning, and participant postitem ratings of perceived difficulty, clarity, and relevance.

The study was designed to address 2 linked questions. First, are human-edited GenAI-assisted MCQs necessarily too easy for postgraduate FM assessment? Second, if such items achieve plausible difficulty, do they demonstrate sufficient discrimination and distractor functioning to support assessment use? By evaluating item functioning rather than relying solely on surface plausibility or expert judgment, this study contributes empirical validity evidence for integrating GenAI into postgraduate MCQ development.

Accordingly, the primary aim of this study was to determine whether human-edited GenAI-assisted MCQs differ from educator-crafted MCQs in postgraduate FM AKT–level psychometric performance. The primary objective was to compare participant total scores between item origins. Secondary objectives were to compare internal consistency reliability, item difficulty, corrected point-biserial discrimination, distractor functioning, and participant postitem ratings of perceived difficulty, clarity, and relevance. We hypothesized that human-edited GenAI-assisted MCQs would not necessarily be easier than educator-crafted MCQs but might show residual vulnerabilities in discrimination and distractor functioning despite educator editing.

## Methods

### Study Design and Setting

We conducted a blinded cross-sectional, within-participant comparative psychometric evaluation of best-of-five single-best-answer MCQs targeted at the postgraduate FM AKT level. The study was conducted in Singapore in the postgraduate FM AKT preparation context after Domain Specific Review Board approval (Ethics and Compliance Online System 2025‐0105). The primary comparative factor was item origin: human-edited GenAI-assisted items versus educator-crafted items.

The study evaluated 60 MCQs in total: 30 human-edited GenAI-assisted items and 30 educator-crafted items. Items were topic-matched at the topic or domain level, with 1 GenAI-assisted item and 1 educator-crafted item developed for each of 30 postgraduate FM topics. The complete topic blueprint is provided in Table S3 in Multimedia Appendix 1. This matching strategy was used to balance curriculum coverage and improve content comparability between item origins. However, matched items were not designed as identical parallel forms and could differ in clinical scenario, decision point, cognitive process, and option structure. Items were organized into 2 assessment sets and presented in randomized order to reduce ordering effects and minimize the likelihood that participants could infer item origin.

The evaluation focused on empirical trainee response data. Outcomes were analyzed at the participant level, test level, item level, distractor level, and response process level. Participant-level outcomes included total scores on GenAI-assisted and educator-crafted items. Test-level outcomes included internal consistency reliability. Item-level outcomes included difficulty and discrimination. Distractor-level outcomes included nonfunctioning distractors and negatively discriminating distractors. Response process outcomes included participant postitem ratings of perceived difficulty, clarity, and relevance to day-to-day FM practice. The overall study design, item origins, matching strategy, blinding, and outcome levels are summarized in Table 1.

**Table 1.** Study design and evaluation framework for a blinded comparative psychometric evaluation of human-edited, GenAI^a^-assisted, and educator-crafted postgraduate FM AKT^b^-level MCQs^c^ in Singapore.

Domain	Description
Study design	Blinded comparative psychometric evaluation
Total MCQs evaluated	60 best-of-five single-best-answer MCQs
Item origins	30 human-edited GenAI-assisted items; 30 educator-crafted items
Matching strategy	Topic- or domain-matched for curriculum coverage, with 1 GenAI-assisted and 1 educator-crafted item per topic; items were not designed as identical parallel forms
Assessment context	Postgraduate FM AKT-level assessment
Presentation	Items randomized across 2 sets
Blinding	Participants blinded to item origin
Participant-level outcomes	Total score on GenAI-assisted items; total score on educator-crafted items
Test-level outcomes	KR-20^d^ internal consistency reliability; Spearman-Brown reliability projection
Item-level outcomes	Difficulty index; corrected point-biserial discrimination
Distractor-level outcomes	Nonfunctioning distractors; negatively discriminating distractors
Response process outcomes	Participant ratings of perceived difficulty, clarity, and relevance

a GenAI: generative artificial intelligence.

b FM AKT: Family Medicine Applied Knowledge Test.

c MCQs: multiple-choice questions.

d KR-20: Kuder-Richardson Formula 20.

### Inclusion and Exclusion

Participants were postgraduate medical doctors preparing for the FM AKT in Singapore. Eligible participants included doctors enrolled in FM residency within 1 of the 3 public health care institutions and/or doctors enrolled in postgraduate FM programs under the College of Family Physicians Singapore. Participants who withdrew consent or did not complete both MCQ sets were excluded from the complete-case analytic sample.

### Sampling Procedures

Participants were recruited by email invitation using convenience sampling with snowball dissemination from January 6 to April 14, 2026, during the 2026 AKT preparation period. Recruitment invitations were disseminated through email, WhatsApp groups, and peer sharing to postgraduate doctors preparing for the FM AKT in Singapore. Participation was voluntary, and participants provided informed consent before data collection.

### Sample Size, Power, and Precision

A priori sample size estimation was performed using G*Power software (version 3.1; Heinrich Heine University Düsseldorf) for the participant-level comparison of total scores between GenAI-assisted and educator-crafted items. As each participant answered both item types, the primary participant-level comparison was planned using a 2-tailed paired-sample *t* test, with α=.05 and power=0.80. Assuming a moderate within-participant standardized effect size of dz=0.40, 52 complete paired participants were required. A smaller effect size of dz=0.30 would require approximately 90 complete paired participants to retain 80% power. We therefore set a recruitment target range of 52-90 participants. The final analytic sample of 73 complete paired responses exceeded the minimum required sample for detecting a moderate paired difference in total scores. Item-level and distractor-level analyses were based on 30 items per origin and were interpreted as exploratory, with emphasis placed on the convergence of findings across difficulty, discrimination, reliability, and distractor-functioning indicators rather than on isolated *P* values.

### Participant Characteristics

Participant characteristics available for analysis were limited to postgraduate training pathway and residency year. Age, sex, years of clinical experience, prior AKT attempt status, and prior GenAI use were not collected in the analytic dataset. Participant characteristics for the complete-case participants are summarized in Table 2.

**Table 2.** Participant characteristics of the 73 postgraduate family medicine (FM) doctors in Singapore included in the complete-case analysis.

Postgraduate program	Values, n (%)	
Graduate diploma in FM	36 (49.3)	
College Master of Medicine in FM program	5 (6.8)	
FM residency year 1	8 (11.0)	
FM residency year 2	21 (28.8)	
FM residency year 3	3 (4.1)	
Total	73 (100)	

### Measures, Covariates, and MCQ Development Workflow

#### Overview

The study materials comprised 60 best-of-five single-best-answer MCQs blueprint-aligned to postgraduate FM AKT–level practice. The primary exposure was item origin: human-edited GenAI-assisted versus educator-crafted. Items sampled clinical domains relevant to FM, including pediatric, adult, and geriatric care, and acute, chronic, preventive, and palliative care. No participant demographic or clinical covariates were included in the primary analyses because the study focused on paired item-origin comparisons and item-level psychometric functioning.

The item-development and review group comprised 3 clinician-educator authors (KPS, JQL, and QWF) and a 5-member expert panel (TYGD, WCW, TC, JLW, and VB) with FM clinical and postgraduate assessment experience. These 8 reviewers provided subject-matter review for clinical accuracy, relevance, and alignment with postgraduate FM standards.

#### Educator-Crafted MCQs

Thirty MCQs were developed by clinician-educators using the study blueprint, a sample AKT-style question, and MCQ-writing guidance (Annex S2 in Multimedia Appendix 1). Educator-crafted items were written to assess applied FM, knowledge, clinical judgment, and guideline-concordant management. Items were reviewed by the study team before inclusion to ensure alignment with the intended topic, answer key, and single-best-answer format.

#### GenAI-Assisted MCQs

Thirty MCQs were generated using ChatGPT-5.1 (OpenAI) [11] and subsequently edited by the study team. GenAI-assisted items were created using tailored prompts that specified the clinical topic, intended learning objective, expected AKT-style single-best-answer format, and level of applied clinical reasoning (Annex S1 in Multimedia Appendix 1). The same sample question and MCQ-writing guidance provided to educator item writers were also provided to the GenAI system.

The GenAI outputs were then reviewed and edited by the same study team for clinical correctness, local guideline contextualization, phrasing, single-best-answer structure, and alignment with intended learning outcomes. Each GenAI-assisted item underwent 1 structured educator review-and-editing round, followed by final study team checking before administration. Editors were not blinded to item origin during item development because the GenAI-assisted items were generated and edited through a prespecified human-in-the-loop workflow; blinding was applied at the participant-assessment stage and not at the item-editing stage. The detailed GenAI-prompting and human-editing framework is provided in Table S2 in Multimedia Appendix 1. The final GenAI-assisted items therefore represent a human-in-the-loop GenAI-assisted workflow, rather than raw artificial intelligence (AI)–generated outputs. The overall human-in-the-loop workflow for developing, reviewing, administering, and evaluating the GenAI-assisted MCQs is summarized in Table 3.

A total of 30 MCQs per item origin were selected pragmatically to balance sufficient content sampling across the FM AKT blueprint with feasibility for both item development and participant testing. A larger item set may have provided more stable item-level psychometric estimates but would have increased educator workload for item writing, review, and standardization, and participant fatigue during test completion. Given that the study was designed as an exploratory blinded psychometric evaluation rather than a high-stakes summative assessment or definitive item calibration study, 30 items per origin were considered an appropriate balance between breadth, feasibility, and response quality.

**Table 3.** Human-in-the-loop workflow^a^ for developing, reviewing, administering, and evaluating GenAI^b^-assisted MCQs^c^.

Stage	Workflow
1. Blueprint and topic selection	AKT^d^ topic, learning objective, and expected reasoning level defined by educators.
2. Structured GenAI prompting	Topic, single-best-answer format, clinical context, sample question, and item-writing guidance supplied to ChatGPT-5.1
3. Draft item generation	Stem, keyed answer, distractors, and explanation generated for educator review
4. Educator review and editing	Clinical accuracy, local guideline concordance, phrasing, key defensibility, and single-best-answer structure checked
5. Option-set engineering	Distractors reviewed for authentic misconceptions, common reasoning errors, homogeneity, cueing, and near-miss plausibility
6. Blinded trainee administration	Randomized items administered to participants blinded to item origin
7. Empirical item analysis	Difficulty, discrimination, reliability, distractor functioning, and participant ratings reviewed
8. Item-bank decision	Accept, revise, quarantine, or retire items based on expert and empirical evidence

a Quality assurance principle: expert-judged item plausibility alone is insufficient; empirical item–functioning evidence is also required.

b GenAI: generative artificial intelligence.

c MCQs: multiple-choice questions.

d AKT: Applied Knowledge Test.

### Quality of Measurements and Instrumentation

Measurement quality was supported through blueprint alignment, topic matching, and use of the same subject-matter review for both item origins. The postitem ratings of perceived difficulty, clarity, and relevance were study-specific 5-point Likert-type items embedded after each MCQ and used as response process evidence rather than as validated psychometric scales. Objective item functioning was evaluated separately using difficulty index, corrected point-biserial discrimination, Kuder-Richardson Formula 20 (KR-20) reliability, and distractor analysis.

### Data Collection and Masking

Participants completed both MCQ sets during the study assessment. Items were presented in randomized order across the 2 sets to reduce ordering effects. Participants were not informed which items were GenAI-assisted or educator-crafted, which served as participant masking to reduce expectancy effects and prevent assumptions about item origin from influencing responses or postitem ratings.

Immediately after answering each MCQ, participants completed brief item-level appraisals. These included ratings of perceived difficulty, clarity of the stem and options, and relevance to day-to-day FM practice. Ratings were collected using embedded Likert-type response options after each question. Participants were also provided with an optional free-text field to flag confusing wording or other item-specific concerns.

### Conditions and Design

This was a nonclinical, noninterventional, and blinded comparative psychometric evaluation. The primary comparison condition was item origin: human-edited GenAI-assisted versus educator-crafted MCQs. Participants were not randomized to intervention groups; all complete-case participants answered both item types, allowing paired participant-level comparisons. Item-level and distractor-level analyses were conducted by item origin and interpreted as exploratory because the topic-matched items were not designed as identical parallel forms.

### Measures and Outcomes

Outcomes were analyzed across 5 levels: participant-level performance, test-level reliability, item-level psychometric functioning, distractor-level functioning, and participant postitem ratings. Definitions and interpretation thresholds for the study outcomes are provided in Table S1 in Multimedia Appendix 1.

#### Participant-Level Outcomes

The primary participant-level outcomes were total score on GenAI-assisted items and total score on educator-crafted items. Each participant therefore contributed paired scores, one for each item origin.

#### Test-Level Outcomes

Internal consistency reliability was calculated separately for GenAI-assisted and educator-crafted item sets using the KR-20. Spearman-Brown prophecy estimates were used to project how reliability would change with longer test length.

#### Item-Level Outcomes

Item difficulty was calculated for each MCQ using the difficulty index, defined as the proportion of participants who answered the item correctly. Higher values indicate easier items. Items were categorized as follows (Table 4).

Item discrimination was assessed using the corrected point-biserial correlation, which correlates performance on an individual item with total test score after excluding that item from the total. Items with corrected point-biserial correlations ≥0.30 were considered to have good discrimination. Negative corrected point-biserial values were treated as red flags because they suggest that higher-performing participants were more likely to answer the item incorrectly.

**Table 4.** Difficulty index thresholds used to classify postgraduate FM AKT^a^-level MCQ^b^ item difficulty in the blinded psychometric evaluation.

Difficulty index	Interpretation
*P*≤.25	Too difficult
.25<*P*<.75	Appropriate difficulty
*P*≥.75	Too easy

a FM AKT: Family Medicine Applied Knowledge Test.

b MCQ: multiple-choice question.

#### Distractor-Level Outcomes

Distractor analysis was conducted for each item. Nonfunctioning distractors were defined as distractors selected by fewer than 5% of participants. Negatively discriminating distractors were defined as distractors with positive point-biserial correlations with total score, indicating that they were selected more frequently by higher-performing participants. For each item, the number of nonfunctioning distractors and negatively discriminating distractors was tallied from 0 to 4.

#### Response-Process Outcomes

Participant postitem ratings were analyzed as response process evidence. These included perceived item difficulty, clarity, and relevance to day-to-day FM practice. These ratings were used to assess whether trainees perceived GenAI-assisted and educator-crafted items differently, and whether subjective ratings aligned with objective item difficulty.

### Data Sources

Data sources comprised primary participant item response and postitem rating data, the answer key, item origin labels, topic or domain mapping, and aggregate psychometric outputs generated from the study datasets. Expert review files informed item development and supplementary materials but were not participant outcome data.

### Data Diagnostics and Missing Data

The analytic dataset used complete paired responses. One participant did not provide complete paired MCQ-set data and was excluded from the complete-case analysis (1/74, 1.4%). The final analytic sample contained no missing item-response data for the 73 included participants, so the Little Missing Completely at Random test and multiple imputation were not performed. Item-level statistics with no response variance were reported as not estimable where applicable.

### Analytic Strategy

Total scores for GenAI-assisted and educator-crafted items were compared using a paired-sample test because each participant completed both item types. The mean difference, 95% CI, *P* value, and Cohen *d* were reported. Pearson correlation coefficient was used to assess the linear relationship between GenAI-assisted and educator-crafted total scores. Bland-Altman analysis was used to assess agreement between the total scores of GenAI-generated and educator-crafted items.

Internal consistency reliability was assessed separately for the GenAI-assisted and educator-crafted item sets using KR-20, which is equivalent to Cronbach α for dichotomously scored items. KR-20 values range from 0 to 1, with higher values indicating greater internal consistency [12]. The 95% CIs for KR-20 were calculated using bootstrap (bias-corrected and accelerated) with 1000 resamples. The Spearman-Brown prophecy formula was used to estimate the number of items that would be required to achieve conventional reliability thresholds, assuming comparable item properties.

Psychometric item analysis was conducted for each MCQ. The difficulty index was calculated as the proportion of participants who answered the item correctly. Corrected point-biserial correlations were calculated to assess item discrimination. Items with no response variance were excluded from point-biserial calculation because discrimination could not be estimated.

Distractor analysis was conducted by item origin. For each item, the number of nonfunctioning distractors and negatively discriminating distractors was counted. Fisher exact tests were used to compare distributions between GenAI-assisted and educator-crafted items because several expected cell counts were below 5.

Participant ratings of perceived difficulty, clarity, and relevance were recoded numerically for analysis. Mean ratings were calculated for each item and compared between GenAI-assisted and educator-crafted items. Correlations between subjective difficulty ratings and objective item difficulty were examined to assess whether participant perceptions aligned with empirical item performance.

Participant-level total score analyses were paired because each participant completed both GenAI-assisted and educator-crafted items. Item-level analyses were conducted by item origin using the 30 items in each group. Independent samples *t* tests were used for item-level comparisons. Although items were matched by clinical topic, GenAI-assisted and educator-crafted items were developed through fundamentally different processes—one by a large language model with subsequent human editing, the other by educators, and topic matching does not guarantee that paired items share residual variance, which is a key assumption underlying paired sample *t* test.

All statistical tests were interpreted at α=.05. Bonferroni correction was applied within each family of comparisons: item-level psychometric analysis, distractor analysis, and participant-rating analysis. For item-level psychometric analyses, 2 tests were conducted, giving a corrected α=.025. For distractor analyses, 2 tests were conducted, giving a corrected α=.025. For participant-rating analyses, 3 tests were conducted, giving a corrected α=.017. Statistical analyses were performed using R (version 4.4.2; R Core Team).

### Ethical Considerations

Ethical approval for human participant research was obtained from the NHG Health Domain Specific Review Board through the Ethics and Compliance Online System (reference 2025‐0105). No ethics exemption was relied on. Participants received study information before participation and provided informed consent before data collection. Participation was voluntary, participants could withdraw without penalty, and no compensation was provided. Study data were analyzed in aggregate after deidentification, and no participant-identifiable information is reported in the paper, figures, tables, or supplementary material. No participant data were entered into the GenAI system during item generation. GenAI was used to generate draft MCQs from study prompts, topic specifications, learning objectives, sample questions, and item-writing guidance. Final items were reviewed and edited by the study team before administration to participants.

## Results

### Participant Flow

**Figure 1.**
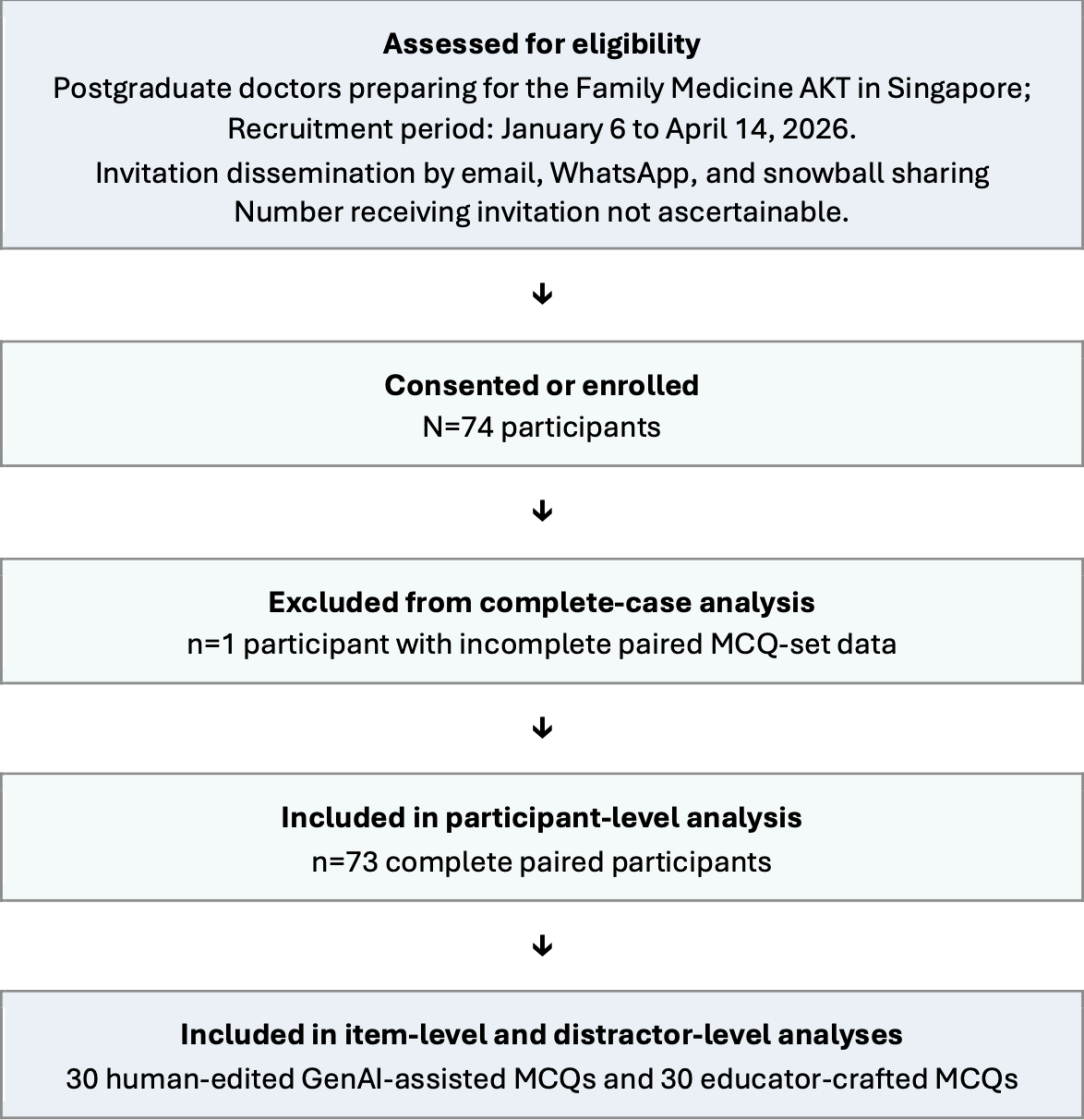
Participant flow diagram for the blinded comparative psychometric evaluation of human-edited GenAI-assisted and educator-crafted postgraduate FM AKT–level MCQs in Singapore. FM AKT: Family Medicine Applied Knowledge Test; GenAI: generative artificial intelligence; MCQ: multiple-choice question.

Recruitment was conducted from January 6 to April 14, 2026, during the 2026 FM AKT preparation period. The total number of doctors who received or viewed the invitation could not be determined because of the snowball recruitment approach. Of the 74 participants who consented and enrolled, 73 completed both MCQ sets and were included in the complete-case participant-level analysis. One participant was excluded because of incomplete paired MCQ-set data. Participant flow is shown in Figure 1.

### Participant-Level Total Scores by Item Origin

Participants scored significantly lower on GenAI-assisted items (mean 19.12, SD 2.83) than on educator-crafted items (mean 21.10, SD 3.42), with a mean paired difference of −1.97 (95% CI −2.72 to −1.23; *P*<.001) and a moderate effect size (Cohen *d*=0.62) (Table 5; Figure S2 in Multimedia Appendix 1). This indicates that the GenAI-assisted item set was more difficult overall than the educator-crafted item set.

**Table 5.** Participant-level and item-level psychometric outcomes by item type in a blinded comparative psychometric evaluation of human-edited GenAI^a^-assisted and educator-crafted postgraduate FM AKT^b^-level MCQs^c^ in Singapore.

Metric	GenAI-assisted, mean (SD)	Educator-crafted, mean (SD)	Mean difference	95% CI	*P* value
Total score	19.12 (2.83)	21.10 (3.42)	−1.97	−2.72 to −1.23	<.001
Difficulty index	0.64 (0.26)	0.70 (0.22)	−0.07	−0.19 to 0.06	.29
Corrected point-biserial correlation	0.09 (0.15)	0.18 (0.15)	−0.08	−0.16 to −0.01	.04^d^

a GenAI: generative artificial intelligence.

b FM AKT: Family Medicine Applied Knowledge Test.

c MCQs: multiple-choice questions.

d Bonferroni-corrected significance thresholds were applied within each family of comparisons: α=.025 for psychometric item analysis (2 tests: difficulty index and corrected point-biserial correlation). The *P* value for corrected point-biserial correlation was below .05 but did not meet the Bonferroni-corrected threshold of α=.025 for this family of comparisons.

### Score Correlation and Agreement

**Figure 2.**
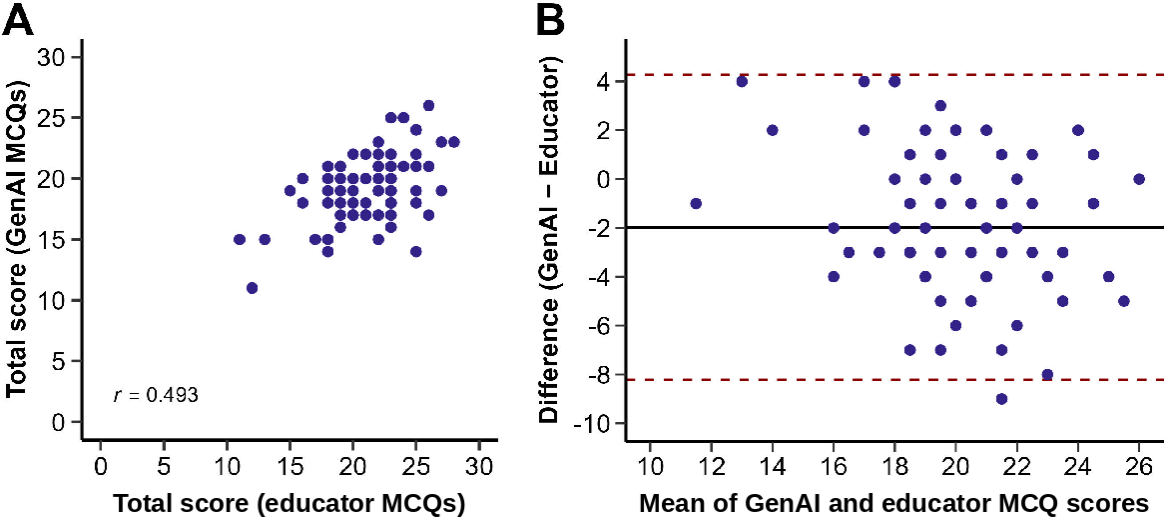
Association and agreement between participants’ total scores for the GenAI-assisted and educator-crafted multiple-choice question sets. (A) Scatterplot showing the correlation between GenAI-assisted and educator-crafted total scores. Each dot represents 1 participant. (B) Bland-Altman plot comparing total test scores of GenAI-assisted and educator-crafted MCQ items. Each dot represents 1 participant. The x-axis shows the mean of the 2 scores; the y-axis shows the difference (GenAI – Educator). The solid line indicates the mean difference, and the dashed lines indicate the 95% limits of agreement. GenAI: generative artificial intelligence; MCQs: multiple-choice questions.

GenAI-assisted and educator-crafted total scores were positively correlated (*r*=0.49, 95% CI 0.30-0.65; *P*<.001) (Figure 2A), indicating that participants who performed well on 1 item set tended to perform well on the other. However, the wide confidence interval—spanning from a weak to moderate association—suggests limited precision in this estimate.

Bland-Altman analysis revealed a mean difference of −1.97 between GenAI-generated and educator-crafted items, with 95% limits of agreement ranging from −8.22 to 4.28 (Figure 2B). This indicates that for any individual participant, their GenAI-assisted MCQ test score could range from approximately 4 points higher to 8 points lower than their educator-crafted score, suggesting that the 2 test forms are not interchangeable at the individual level. Visual inspection suggested possible proportional bias: lower-scoring participants showed score differences closer to zero, while higher-scoring participants showed larger negative differences, suggesting that the GenAI-assisted items disproportionately penalized higher-performing participants.

### Item Difficulty and Discrimination

**Figure 3.**
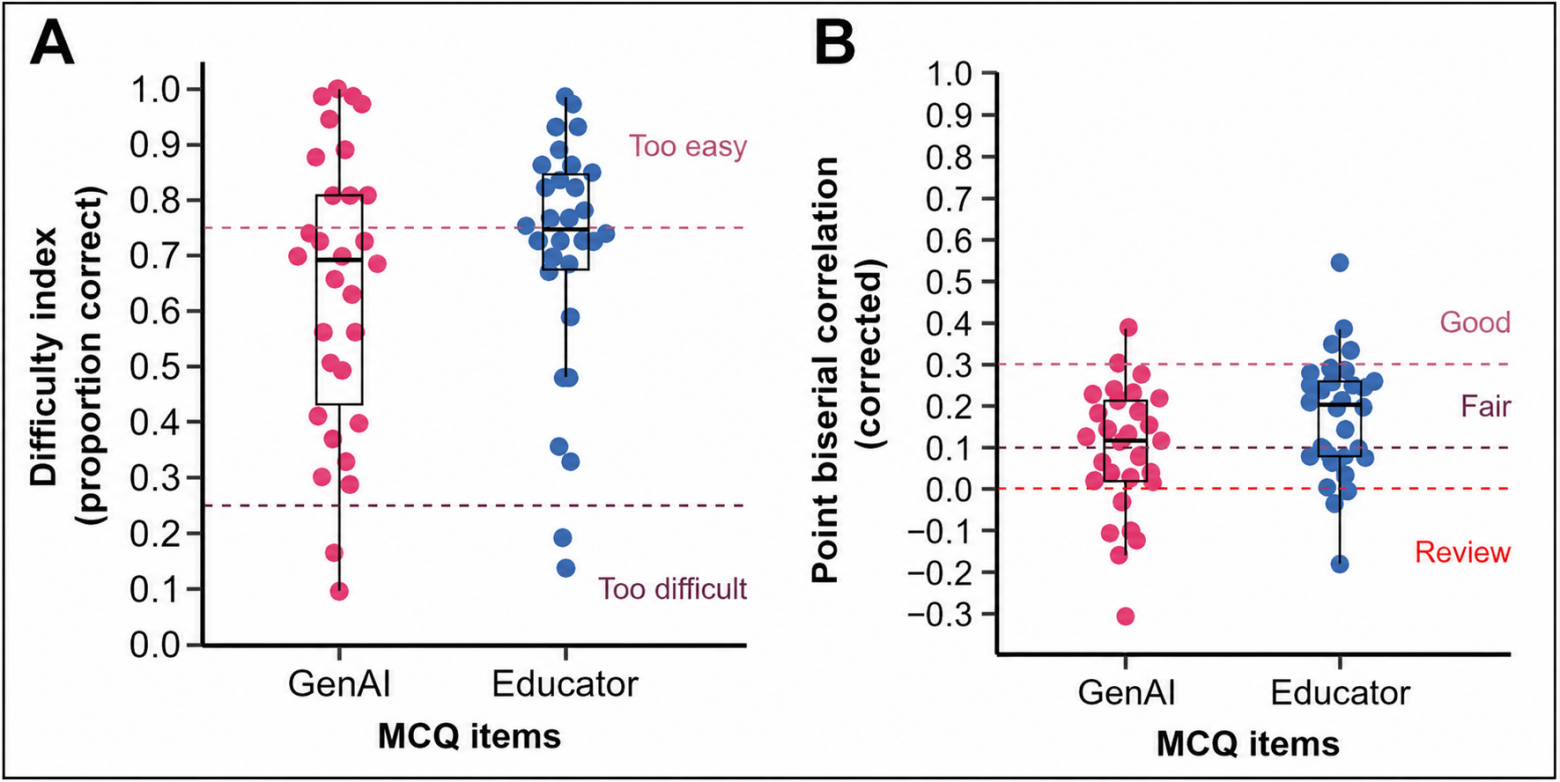
Item difficulty and discrimination by item origin for 30 human-edited GenAI-assisted and 30 educator-crafted postgraduate Family Medicine Applied Knowledge Test–level MCQs in Singapore. (A) Item difficulty measured by difficulty index; higher values indicate easier items. Dashed lines show the acceptable difficulty range of 0.25-0.75. (B) Corrected point-biserial discrimination; higher values indicate stronger discrimination. Dashed lines indicate thresholds for good, fair, and review-level discrimination. Each dot represents 1 item. GenAI: generative artificial intelligence; MCQ: multiple-choice question.

At the item level, GenAI-assisted items were slightly more difficult on average than educator-crafted items, although this difference was not statistically significant. The mean difficulty index was 0.64 (SD 0.26) for GenAI-assisted items and 0.70 (SD 0.22) for educator-crafted items, with a mean difference of −0.07 (95% CI −0.19 to 0.06; *P*=.29; Cohen *d*=−0.28) (Table 5 and Figure 3A). Because higher difficulty index values indicate easier items, this item-level finding was consistent with the participant-level score comparison.

When items were classified by difficulty range, a larger proportion of GenAI-assisted items fell within the acceptable difficulty range of 0.25- 0.75. Eighteen GenAI-assisted items (60.0%) were classified as having appropriate difficulty, compared with 13 educator-crafted items (43.3%). Conversely, 10 GenAI-assisted items (33.3%) and 15 educator-crafted items (50.0%) were classified as too easy. Two items from each set (6.7%) were classified as too difficult. One GenAI-assisted item on diabetes mellitus was answered correctly by all participants; because there was no response variance, its corrected point-biserial correlation could not be computed.

Despite achieving plausible difficulty, GenAI-assisted items showed weaker discrimination. The mean corrected point-biserial correlation was 0.09 (SD 0.15) for GenAI-assisted items and 0.18 (SD 0.15) for educator-crafted items. The mean difference was −0.08 (95% CI −0.16 to −0.01; *P*=.04; Cohen *d*=−0.56), which was below 0.05 but did not meet the Bonferroni-corrected threshold of α=.025 for this family of comparisons (Table 5 and Figure 3B).

Only 2 GenAI-assisted items (6.7%) achieved good discrimination, defined as corrected point-biserial correlation ≥0.30, compared with 4 educator-crafted items (13.3%). More concerningly, 6 GenAI-assisted items (20.0%) had negative corrected point-biserial correlations, compared with 3 educator-crafted items (10.0%). Negative corrected point-biserial values indicate that higher-performing participants were more likely to answer these items incorrectly, suggesting that these items require review before reuse. Complete item-level psychometric results by topic and item source are provided in Table S4 in Multimedia Appendix 1.

### Internal Consistency Reliability

Both item sets demonstrated suboptimal internal consistency reliability, although reliability was lower for GenAI-assisted items (KR-20=0.38, 95% CI 0.12-0.56) than for educator-crafted items (KR-20=0.60, 95% CI 0.44-0.73).

Alpha-if-item-deleted analysis showed that removing 1 GenAI-assisted ethics item increased the GenAI-assisted KR-20 from 0.38 to 0.45, an increase of approximately 18%. No single educator-crafted item had a comparable effect on the educator-crafted item set reliability (Figure S3 in Multimedia Appendix 1).

As internal consistency reliability is influenced by test length, the Spearman-Brown prophecy formula was used to project reliability for longer assessments. Assuming comparable item properties, a 200-item test would increase projected KR-20 to 0.80 for GenAI-assisted items and 0.91 for educator-crafted items. To achieve a KR-20 of 0.70, an estimated 116 GenAI-assisted items and 48 educator-crafted items would be required. These projections should be interpreted as illustrative because they assume that additional items would have similar psychometric properties to those observed in this study.

### Distractor Functioning

Distractor analysis was conducted to explore whether option-set quality contributed to weaker discrimination among GenAI-assisted items. A higher proportion of GenAI-assisted items had multiple nonfunctioning distractors and negatively discriminating distractors, although these comparisons did not reach statistical significance (Fisher exact *P*=.46 and *P*=.41, respectively).

**Figure 4.**
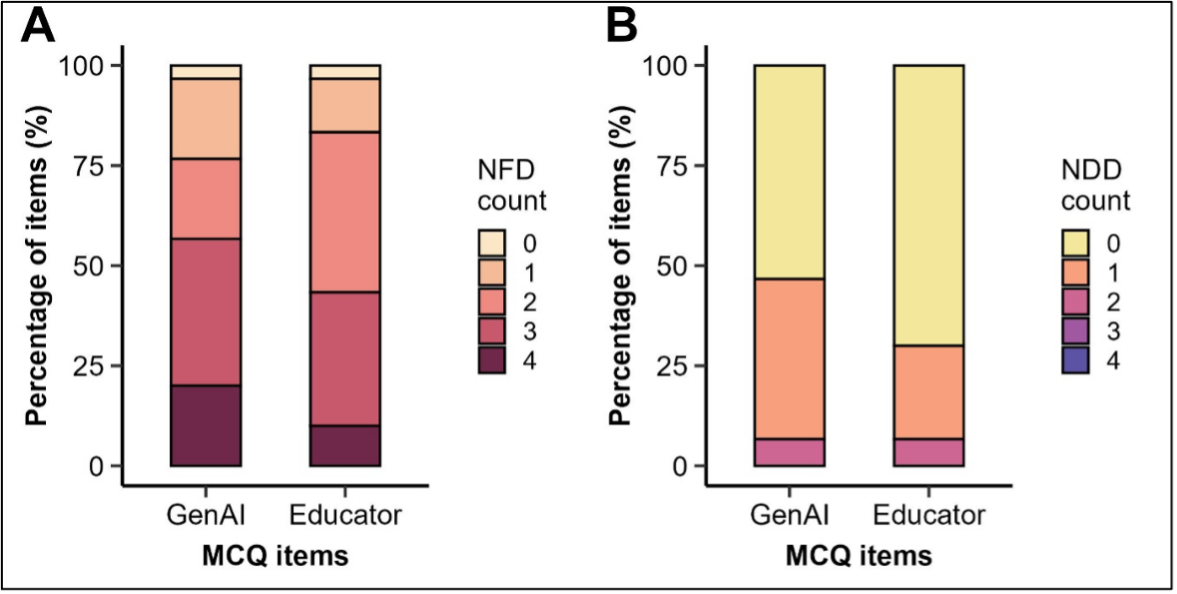
Distractor-functioning red flags by item origin for 30 human-edited GenAI-assisted and 30 educator-crafted postgraduate Family Medicine Applied Knowledge Test–level MCQs in Singapore. (A) Distribution of nonfunctioning distractors per item; nonfunctioning distractors were selected by fewer than 5% of participants. (B) Distribution of negatively discriminating distractors per item; negatively discriminating distractors were selected more frequently by higher-performing participants. Shading indicates the number of problematic distractors per item. GenAI: generative artificial intelligence; MCQ: multiple-choice question; NDD: negatively discriminating distractor; NFD: nonfunctioning distractor.

Seventeen GenAI-assisted items (56.7%) had 3 or 4 nonfunctioning distractors, compared with 13 educator-crafted items (43.3%) (Table 6 and Figure 4A). Fourteen GenAI-assisted items (46.7%) contained 1 or 2 negatively discriminating distractors, compared with 9 educator-crafted items (30.0%) (Table 6 and Figure 4B).

Items with negative corrected point-biserial correlations frequently co-occurred with poor distractor functioning. Among the 6 GenAI-assisted items with negative corrected point-biserial correlations, 5 had at least 3 nonfunctioning distractors and 4 had 1 or 2 negatively discriminating distractors. Among the 3 educator-crafted items with negative corrected point-biserial correlations, 1 had 3 nonfunctioning distractors and 2 had 1 or 2 negatively discriminating distractors. This pattern suggests that weak discrimination may be partly explained by option-set problems, particularly distractors that were either rarely selected or selected disproportionately by higher-performing participants.

**Table 6.** Item classification and distractor-functioning red flags by item origin for 30 human-edited GenAI^a^-assisted and 30 educator-crafted postgraduate FM AKT^b^–level MCQs^c^ in Singapore.

Item-level classification	GenAI-assisted, n (%)	Educator-crafted, n (%)
Appropriate difficulty, 0.25<*P*<.75	18 (60)	13 (43.3)
Too easy, *P*≥.75	10 (33.3)	15 (50)
Too difficult, *P*≤.25	2 (6.7)	2 (6.7)
Good discrimination, rpb(c)^d^≥0.30	2 (6.7)	4 (13.3)
Negative discrimination, rpb(c)<0	6 (20)	3 (10)
3 or 4 nonfunctioning distractors	17 (56.7)	13 (43.3)
1 or 2 negatively discriminating distractors	14 (46.7)	9 (30)

a GenAI: generative artificial intelligence.

b FM AKT: Family Medicine Applied Knowledge Test.

c MCQs: multiple-choice questions.

d rpb(c): corrected point-biserial correlation coefficient.

### Participant Postitem Ratings

Participant postitem ratings did not differ significantly between GenAI-assisted and educator-crafted items. Mean ratings showed no significant difference by item origin for perceived difficulty (mean difference 0.06, 95% CI −0.12 to 0.23; *P*=.51), clarity (mean difference −0.03, 95% CI −0.08 to 0.03; *P*=.38), or relevance to day-to-day FM practice (mean difference 0.08, 95% CI −0.05 to 0.22; *P*=.20). None of these comparisons met the Bonferroni-corrected threshold of α=.017 for the participant-rating analysis family.

Subjective difficulty ratings were associated with objective item difficulty. For both GenAI-assisted items and educator-crafted items, mean perceived difficulty ratings were negatively correlated with difficulty index (*r*=−0.72, 95% CI −0.86 to −0.49; *P*<.001 and *r*=−0.54, 95% CI −0.76 to −0.23; *P*=.002, respectively), indicating that items rated as harder were answered correctly by fewer participants (Figure S4 in Multimedia Appendix 1). These findings suggest that participants did not perceive GenAI-assisted items as systematically less clear, less relevant, or more difficult than educator-crafted items.

## Discussion

### Principal Findings

This blinded comparative psychometric evaluation provides practice-relevant evidence on the use of human-edited GenAI-assisted MCQs, rather than raw GenAI outputs, in a postgraduate FM AKT–level context. The central finding is that GenAI-assisted items were not easier than educator-crafted items. Participants scored significantly lower on the GenAI-assisted item set, mean item difficulty did not differ significantly between item origins, and a greater proportion of GenAI-assisted items fell within the acceptable difficulty range. These findings challenge the assumption that GenAI-assisted MCQs are inherently too easy when they are generated using AKT-aligned prompts and subsequently edited by educators.

However, plausible difficulty did not translate into equivalent assessment functioning. Compared with educator-crafted items, GenAI-assisted items showed lower internal consistency reliability, weaker corrected point-biserial discrimination, fewer items with good discrimination, more items with negative discrimination, and more distractor-functioning red flags. Although several item-level comparisons did not meet Bonferroni-corrected thresholds, the direction of findings was consistent across complementary indicators. The interpretation of borderline findings in this study warrants explicit discussion. The corrected point-biserial discrimination comparison yielded *P*=.04, which fell below the conventional α=.05 threshold but did not meet the Bonferroni-corrected threshold of α=.025 for the psychometric item analysis family. Bonferroni correction was applied to reduce the risk of false positives across multiple comparisons, which is statistically appropriate. However, because the study was not powered specifically for all item-level comparisons, this conservative adjustment also increases the risk of overlooking genuine differences, particularly where effect sizes are small to moderate and item samples are limited to 30 per group. The mean difference in corrected point-biserial correlation of −0.08 (95% CI −0.16 to −0.01) represents a clinically and educationally meaningful gap, given that the GenAI-assisted mean of 0.09 falls in the poor to marginal range while the educator-crafted mean of 0.18 approaches the acceptable range. Rather than treating this finding as simply nonsignificant, we interpret it as a borderline result that is consistent in direction with the broader pattern of weaker reliability, more negative discrimination signals, and more distractor-functioning concerns observed across GenAI-assisted items. Taken together, the convergence of findings across multiple complementary indicators provides stronger cumulative support for the interpretation of residual psychometric vulnerability than any single comparison in isolation. These results suggest that the key concern with edited GenAI-assisted MCQs was not difficulty targeting but whether the items could reliably distinguish stronger from weaker candidates and whether distractors functioned as intended. Participant postitem ratings add a further important layer. Trainees did not perceive GenAI-assisted items as systematically less clear, less relevant, or more difficult than educator-crafted items. Subjective difficulty ratings also correlated with objective item difficulty, suggesting that participant ratings provided meaningful response process information. However, similar participant ratings across item origins did not preclude differences in psychometric functioning, including reliability, discrimination, and distractor performance.

Both item sets demonstrated suboptimal internal consistency reliability, which is partly attributable to the relatively small number of items in each set (30 items). Spearman-Brown projections suggest that longer sets would achieve acceptable to good reliability; assuming comparable item properties, a 200-item test would increase projected KR-20 to 0.80 for GenAI-assisted items and 0.91 for educator-crafted items [13]. However, the GenAI-assisted set would still require substantially more items than the educator-crafted set to reach KR-20=0.70, reinforcing the need for targeted item repair before use in formal assessment.

The low internal consistency of the GenAI-assisted items and their weaker item discrimination are psychometrically interrelated. A noisy total score serves as a less reliable anchor for point-biserial correlations, which may attenuate observed discrimination values. However, several findings from this study suggest that intrinsic item flaws are the primary driver, rather than a systemic symptom of lower internal consistency. First, distractor analysis revealed that many GenAI-assisted items contained nonfunctional or negatively discriminating distractors, indicating structural problems at the item level. Second, alpha-if-deleted analysis identified specific items whose removal improved KR-20, suggesting that particular problematic items depressed overall reliability rather than the reverse. Together, these findings indicate that weak discrimination stems primarily from deficiencies in distractor construction. Wide confidence intervals were observed across several key metrics, including item-level comparisons, correlations, and KR-20, reflecting the limited number of items (30 per group), which constrain the precision of between-group comparisons and strength of associations.

### Comparison With Prior Work

Our finding that human-edited GenAI-assisted items were not empirically easier than educator-crafted items contrasts with several recent high-stakes and residency-based comparisons in which AI-generated items tended to be easier on average [2,7]. This difference may reflect our focus on a human-in-the-loop workflow with AKT-aligned prompting and educator editing prior to administration, which likely mitigates the “easy-item” tendency reported when AI outputs are used with less structured refinement [8]. At the same time, our broader pattern of weaker reliability, lower discrimination, and more distractor-functioning red flags despite similar perceived clarity and relevance aligns with emerging evidence that surface plausibility and perceived quality do not guarantee psychometric readiness, and that distractor construction remains a persistent vulnerability in AI-assisted item writing [9,10]. Dhanvijay et al [14] similarly reported that AI-chatbot–generated undergraduate physiology MCQs contained more nonfunctioning distractors than faculty-written MCQs, but their AI items were easier and showed comparable discrimination rather than weaker discrimination. This contrast is useful because it suggests that distractor vulnerability may recur across contexts, while difficulty and discrimination patterns may depend on discipline, learner group, item source, and human-review workflow. Studies that report broadly comparable psychometric properties or detectability across AI and human items have typically emphasized the role of expert review and structured workflows [4-6], consistent with our interpretation that GenAI is best positioned as a drafting adjunct rather than an autonomous item writer. Taken together, the literature suggests that the key implementation question is shifting from whether GenAI are “too easy” to whether postediting and option-set engineering are sufficient to ensure discrimination, defensible keys, and functioning distractors at scale. Our study adds practice-relevant validity evidence for this question by evaluating edited GenAI-assisted items using trainee response data and distractor-level diagnostics in a broad postgraduate FM assessment context.

### Interpretation and Limitations

#### GenAI-Assisted MCQs Can Achieve Difficulty, but Difficulty Is Not Validity

A key implication of this study is that GenAI-assisted MCQs should not be dismissed as inevitably easy or purely recall-based. In our postgraduate FM context, edited GenAI-assisted items were answered correctly less often than educator-crafted items, and more of them fell within the acceptable difficulty range. This suggests that, with structured prompting and human editing, GenAI-assisted workflows can produce items that are challenging enough for postgraduate learners.

However, empirical difficulty should not be mistaken for validity. Difficulty indicates how many candidates answered an item correctly; it does not show whether the item measures the intended construct, whether the keyed answer is uniquely defensible, or whether the item distinguishes stronger from weaker candidates. A recall item can be difficult if it tests obscure information, while an applied clinical item can be easy if the correct management decision is obvious. Conversely, a difficult item can still be psychometrically poor if it is ambiguous or dependent on distractors that do not function as intended [15-18].

This distinction is particularly important for GenAI-assisted item development. Large language models can generate fluent and clinically plausible MCQ stems, and human editing can improve factual correctness, local guideline alignment, and phrasing. Yet, these steps do not necessarily establish that the final item contributes to defensible score interpretation. In this study, GenAI-assisted items achieved plausible difficulty but showed weaker internal consistency and discrimination. The practical lesson is therefore not that GenAI cannot generate sufficiently challenging MCQs. Rather, the lesson is that appropriate difficulty is necessary but insufficient evidence of assessment readiness.

This also reframes how educators should interpret the current literature. Concerns that GenAI-generated MCQs may be too easy or lower-order remain important, but our findings suggest that difficulty may be a solvable problem when prompts are aligned to postgraduate assessment expectations and outputs are human-edited. The more persistent threat may lie downstream in whether the item functions psychometrically once administered to the target learner population [8-10].

#### Distractor Functioning as a Residual Vulnerability

The distractor analysis is consistent with, but does not prove, the interpretation that option-set problems contributed to weaker discrimination among GenAI-assisted items. A higher proportion of GenAI-assisted items contained multiple nonfunctioning distractors and 1 or 2 negatively discriminating distractors. Items with negative corrected point-biserial correlations frequently co-occurred with poor distractor functioning. These patterns co-occurred, but the current data cannot establish directionality. Poorly functioning distractors may weaken discrimination, but broader item flaws such as ambiguous wording, contestable keys, or construct-irrelevant complexity may also produce both weak discrimination and unusual distractor selection patterns.

This finding is important because distractors are not decorative. In a well-constructed single-best-answer MCQ, distractors should represent plausible but incorrect alternatives that reflect common misconceptions, reasoning errors, or near-miss clinical decisions. Effective distractors help distinguish candidates who can apply the intended concept from those who cannot. By contrast, nonfunctioning distractors contribute little to measurement precision because few candidates select them. Negatively discriminating distractors are more concerning because they may be selected disproportionately by higher-performing candidates, suggesting ambiguity, competing interpretations, or construct-irrelevant complexity [19,20].

GenAI may be particularly vulnerable at this level. A model can produce distractors that are fluent, grammatically consistent, and superficially plausible, but effective distractor design requires more than surface plausibility. It requires knowledge of how learners commonly misunderstand a concept, which diagnoses or management options are genuinely tempting in practice, and where clinically meaningful decision boundaries lie. These are pedagogical and assessment-design judgments and not simply language generation tasks [19,20].

This possibility may help explain why routine human editing did not fully eliminate psychometric vulnerabilities. Editors may naturally focus on whether the stem is clinically correct, whether the keyed answer is defensible, and whether the wording is clear. Those are essential steps, but they may not be sufficient. Our findings suggest that GenAI-assisted MCQ workflows require deliberate option-set engineering: each distractor should be reviewed for misconception fidelity, clinical plausibility, homogeneity with the key, absence of cueing, and expected diagnostic value. Items with negative discrimination or multiple nonfunctioning distractors should be revised, quarantined, or retired rather than banked unchanged. Post hoc review and repair of poorly discriminating items have been recommended as a mechanism for improving assessment reliability and defensibility [21].

#### Implications for Human-in-the-Loop MCQ Development

These findings support a pragmatic model in which GenAI is used as a rapid drafting adjunct, while educators retain responsibility for assessment design, validity evidence, and final item-bank decisions. The human role should not be limited to proofreading or checking factual correctness. Instead, the highest-yield human contribution may be targeted repair of the option set and postadministration review of item functioning. This places GenAI-assisted item development within an educator-led validity and quality assurance workflow rather than treating AI output as assessment-ready evidence [15,16].

A practical human-in-the-loop workflow would include the following stages. First, educators should define the blueprint area, learning objective, expected cognitive level, and clinical context before prompting GenAI. Second, GenAI can be used to generate draft stems, keys, distractors, and explanations. Third, educators should review the draft for clinical correctness, local guideline concordance, clarity, and single-best-answer structure. Fourth, and most importantly, educators should conduct deliberate distractor engineering, ensuring that distractors represent authentic misconceptions, common clinical reasoning errors, or realistic near-miss decisions. When reviewing GenAI-produced distractors, editors should look specifically for obviously implausible options, repeated grammatical or structural patterns that cue the answer, alternatives that are not homogeneous with the key, and near-miss options that are so broad or ambiguous that knowledgeable candidates may reasonably choose them. Such distractors should be rewritten to target a defined misconception or clinical reasoning error, or removed if no plausible misconception can be identified. Fifth, items should be piloted with the intended learner population where feasible. Finally, empirical item analysis should guide whether items are accepted, revised, quarantined, or retired. The emphasis on distractor engineering aligns with established principles that distractors should be plausible, homogeneous, and educationally meaningful rather than merely grammatically plausible alternatives [19,20].

In this workflow, negative corrected point-biserial values should be treated as a serious red flag and should preclude item reuse without careful review of the construct, wording, key, and distractors. Similarly, items with multiple nonfunctioning distractors should be revised even if the stem appears clinically reasonable and the item difficulty falls within an acceptable range. For GenAI-assisted items in particular, the threshold for empirical review should be especially explicit, as surface fluency may create a false sense of readiness. Poor or negative discrimination should therefore prompt conservative item review because it may indicate ambiguity, keying problems, or construct-irrelevant complexity [17,18].

Participant ratings can still be useful, but they should be interpreted as supplementary rather than decisive. In this study, perceived difficulty correlated with objective difficulty, suggesting that trainee ratings may help identify items that feel unexpectedly easy, difficult, or confusing. However, ratings of clarity, relevance, and perceived difficulty were similar between GenAI-assisted and educator-crafted items, although the 2-item sets showed different patterns of reliability, discrimination, and distractor functioning. Therefore, learner ratings may support triage, but they should not replace psychometric analysis or expert review.

The broader implication is that GenAI changes the educator’s task. The educator is no longer only an item writer starting from a blank page. The educator becomes a validity-oriented editor and item-quality steward, responsible for transforming fluent drafts into assessment-ready items through construct alignment, distractor repair, pilot testing, and empirical review [15,16].

Future research should also pair psychometric evaluation with workflow and efficiency data, including the time required for prompting, educator editing, distractor repair, expert review, and post–pilot revision. This would help determine whether GenAI-assisted item development improves not only item-bank scalability but also the efficiency of producing assessment-ready MCQs.

#### Formative and Summative Assessment Implications

The threshold for using GenAI-assisted MCQs should differ between formative and summative contexts. For formative education, edited GenAI-assisted MCQs may be valuable before they reach the psychometric standard required for high-stakes testing, provided the questions are labeled and used as learning resources rather than evidence of competence. They can expand practice question banks, stimulate discussion, support retrieval practice, and expose trainees to a broader range of clinical scenarios. In formative settings, imperfect items can still have educational value if they are reviewed for factual accuracy and accompanied by clear explanations. However, educators should remain alert to misleading distractors, ambiguous keys, or explanations that reinforce incorrect reasoning [19,20,22].

For summative assessment, the threshold should be substantially higher. Items should not enter an operational examination bank solely because they are clinically plausible, appropriately difficult, or rated favorably by trainees. Summative items should demonstrate acceptable discrimination, functioning distractors, key defensibility, and contribution to internal consistency reliability. Items with negative discrimination should be excluded or repaired before reuse. Items with several nonfunctioning distractors should be revised because they may reduce measurement precision. In high-stakes or progression-related contexts, empirical item analysis is not optional quality enhancement; it forms part of the validity argument supporting defensible score interpretation [15-17].

A reasonable implementation stance is therefore staged adoption. GenAI-assisted items can first be used in low-stakes formative settings after educator review. Items that perform well after pilot administration can then be considered for supervised inclusion in item banks. Only items with satisfactory psychometric performance and expert review should be considered for summative use. This staged approach allows programs to benefit from GenAI’s efficiency while preserving assessment quality and fairness.

### Limitations

This study has several limitations. First, each item set contained only 30 MCQs. While this allowed pragmatic comparison while limiting educator and participant burden, the relatively small number of items limits the precision of item-level psychometric estimates, particularly for discrimination indices, distractor-functioning analysis, and subgroup comparisons. The findings should therefore be interpreted as exploratory and hypothesis-generating. Future studies with larger item banks and repeated testing across cohorts would allow more stable item calibration and stronger inferences about the comparative performance of GenAI-assisted and educator-crafted MCQs.

Second, items were topic- or domain-matched at the design stage rather than analyzed using a formal matched-pair model. Topic matching strengthened content comparability by ensuring that each item origin covered the same broad set of postgraduate FM domains. However, the paired items were not identical parallel forms and could differ in clinical scenario, cognitive process, decision point, and option structure. We therefore treated item-level comparisons as exploratory and did not conduct paired item-level or mixed-effects sensitivity analyses accounting for topic pair. Future studies should prespecify paired item-level analyses or response-level mixed-effects models with participant, item, and topic-pair effects, ideally using larger item pools per topic.

Third, a deliberate design choice was to evaluate human-edited GenAI-assisted items rather than raw GenAI outputs. This reflects the practical reality of GenAI adoption in assessment development, where educators would be expected to review and edit GenAI-generated drafts before use, particularly in postgraduate or high-stakes contexts. The findings should therefore be interpreted as evidence about a human-in-the-loop workflow, rather than autonomous GenAI item generation. While this design does not isolate the independent contribution of prompting, model output, educator editing, and final item selection, it improves ecological validity and tests whether routine human editing is sufficient to produce psychometrically ready items. Future studies could compare raw GenAI outputs, routinely edited GenAI-assisted items, and deliberately distractor-engineered items to identify which quality assurance steps most improve psychometric functioning. Fourth, the study did not include formal expert-panel coding of cognitive level, such as Bloom’s taxonomy or another cognitive-process framework. The findings directly challenge the claim that GenAI-assisted items are necessarily easier because difficulty was empirically measured using trainee response data. However, difficulty and cognitive level are not identical constructs. A difficult item may still test recall, and an applied item may be easy. Future work should combine psychometric analysis with blinded expert coding of cognitive level, item-writing flaws, construct alignment, and distractor quality.

Finally, participant recruitment used convenience sampling among postgraduate FM doctors preparing for the Singapore AKT. The findings may not generalize to undergraduate learners, other specialties, other health care systems, other item formats, or operational high-stakes examinations. Nevertheless, the study provides contextually relevant evidence from a broad, safety-critical postgraduate primary care assessment setting in which GenAI-assisted item development is likely to be increasingly considered.

### Conclusions

Human-edited GenAI-assisted MCQs can achieve plausible and often appropriate difficulty for postgraduate FM assessment, challenging the assumption that such items are necessarily too easy. However, difficulty did not ensure assessment readiness. Compared with educator-crafted items, GenAI-assisted items showed lower internal consistency, weaker discrimination, more negative discrimination signals, and more distractor-functioning concerns, despite similar participant ratings of clarity, relevance, and perceived difficulty. These findings suggest that human editing for accuracy and phrasing does not necessarily eliminate downstream psychometric vulnerabilities. GenAI is therefore best positioned as a rapid drafting adjunct within an educator-led workflow that prioritizes key verification, distractor engineering, empirical item analysis, and conservative repair or removal of poorly functioning items before incorporation into question banks or use in formal assessments.

## Supplementary material

10.2196/100179 Multimedia Appendix 1 Additional methodological definitions, workflow documentation, topic-matching information, sensitivity-analysis specifications, and supplementary figures.

## Abbreviations

AI: artificial intelligence
FM: family medicine
FM AKT: Family Medicine Applied Knowledge Test
GenAI: generative artificial intelligence
KR-20: Kuder-Richardson Formula 20
MCQ: multiple-choice question
