# Supplementary material for: Human-Edited Generative AI-Assisted Multiple-Choice Questions in Postgraduate Family Medicine: Blinded Cross-Sectional Comparative Psychometric Study
Source: JMIR Med Educ. 2026 Aug 10;12:e100179. doi: 10.2196/100179 (PMC13456530; doi:10.2196/100179)

Multimedia Appendix 1

Human-Edited Generative AI-Assisted Multiple-Choice Questions in Postgraduate Family Medicine: A Blinded Cross-Sectional Comparative Psychometric Evaluation

Table S1. Outcome definitions and interpretation thresholds

Outcome	Unit of analysis	Definition	Interpretation or threshold
Total score	Participant	Number of correct responses for each item origin.	Higher score indicates better performance.
KR-20	Item set	Internal consistency reliability for dichotomously scored items.	Higher value indicates greater internal consistency.
Spearman-Brown projection	Item set	Estimated reliability after increasing test length.	Used to estimate the number of items required to reach target reliability.
Difficulty index, P	Item	Proportion of participants answering the item correctly.	Higher value indicates an easier item.
Too difficult	Item	Difficulty index P <= 0.25.	Few participants answered correctly.
Appropriate difficulty	Item	Difficulty index 0.25 < P < 0.75.	Item falls within acceptable difficulty range.
Too easy	Item	Difficulty index P >= 0.75.	Many participants answered correctly.
Corrected point-biserial correlation, rpb(c)	Item	Correlation between item score and total score after excluding that item from the total score.	Higher value indicates stronger discrimination.
Good discrimination	Item	Corrected point-biserial correlation rpb(c) >= 0.30.	Item discriminates well between stronger and weaker candidates.
Negative discrimination	Item	Corrected point-biserial correlation rpb(c) < 0.	Higher-performing participants were more likely to answer incorrectly; item warrants review.

Outcome	Unit of analysis	Definition	Interpretation or threshold
Non-functioning distractor	Distractor	Distractor selected by fewer than 5% of participants.	May indicate an implausible or low-value distractor.
Negatively discriminating distractor	Distractor	Distractor selected more often by higher-performing participants.	May indicate ambiguity, competing interpretation, or keying issue.
Participant ratings	Item	Post-item ratings of perceived difficulty, clarity, and relevance.	Response-process evidence; supplementary to psychometric analysis.

Table S2. GenAI prompting and human editing framework

Workflow stage	Inputs or criteria	Purpose
Topic specification	Clinical topic from the postgraduate Family Medicine AKT blueprint.	Ensures content alignment.
Learning objective	Intended clinical knowledge or decision-making outcome.	Ensures construct alignment.
Item format	Best-of-five single-best-answer MCQ.	Ensures consistency with AKT-style assessment.
Sample question	AKT-style exemplar provided to both the GenAI system and educator item writers.	Standardises expected format.
Item-writing guidance	Same MCQ-writing guidance provided to GenAI prompting workflow and educator item writers.	Improves fairness of comparison.
GenAI draft generation	Stem, key, distractors, and explanation generated from structured prompts.	Produces the initial item draft.
Clinical review	Factual accuracy, local guideline concordance, and safe management.	Prevents clinically incorrect or unsafe items.
Structural review	Single-best-answer structure, clarity, phrasing, and absence of obvious cueing.	Improves item-writing quality.

Workflow stage	Inputs or criteria	Purpose
Option-set review	Plausible distractors, authentic misconceptions, realistic near-miss decisions.	Strengthens discrimination potential.
Final inclusion review	Topic alignment, answer key, clinical plausibility, and readiness for blinded administration.	Determines whether item proceeds to trainee administration.

Table S3. Topic blueprint

Topic No.	Matched topic/domain
1	Endocrine - Neck lump
2	Urology - Urinary tract infection
3	Neonatal jaundice
4	Neurology - Headache
5	Cervical cancer screening
6	Diabetes mellitus – Screening, newly diagnosed
7	Neurology - Peripheral neuropathy
8	Gastrointestinal - Dyspepsia
9	Dermatology - Eczema
10	Respiratory - Chronic obstructive pulmonary disease
11	Psychiatry - Mood disorder
12	Musculoskeletal - Rotator cuff
13	Female reproductive health - Emergency contraception
14	Hematology - Coagulopathy
15	Ethics
16	Diabetes mellitus – Poorly controlled, diabetic ketoacidosis
17	Preventive health - Travel medicine
18	Preventive health - Smoking cessation
19	Cardiovascular - Heart failure
20	Biostatistics
21	Sexually transmitted infections
22	Endocrine - Osteoporosis
23	Cardiovascular - Cardiac murmurs
24	Emerging infections
25	Cardiovascular - Chronic venous insufficiency
26	Orthopaedics - Wrist or hand fracture
27	Renal - Nephrotic syndrome
28	Immune-mediated rheumatic disease
29	Dermatology - Scabies
30	Gastrointestinal - Abnormal liver function test

Topic labels correspond to the matched item-pair structure used for analysis.

Table S4: Item-Level Psychometric Performance by Topic and Item Source							
**Item**	**Topic**	**Type**	**Difficulty Index (P)**	**Point Biserial Correlation (corrected)**	**Difficulty Index category**	**Point Biserial Correlation category**	**KR-20-if-item-deleted**
Question_16_A_AI	Diabetes Mellitus – Screening, Newly diagnosed	AI	1.000	NA	Too easy	NA	NA
Question_23_A_AI	Dermatological - Scabies	AI	0.986	0.303	Too easy	Good	0.362
Question_8_B_AI	Diabetes Mellitus – Poorly controlled, Diabetic ketoacidosis	AI	0.986	-0.161	Too easy	Review	0.388
Question_18_B_AI	Neurological - Peripheral neuropathy	AI	0.973	0.039	Too easy	Poor	0.377
Question_30_A_AI	Gastrointestinal - Dyspepsia	AI	0.945	0.212	Too easy	Fair	0.358
Question_14_A_AI	Endocrine: Neck Lump	AI	0.890	0.077	Too easy	Poor	0.372
Question_20_A_AI	Neonatal jaundice	AI	0.877	0.063	Too easy	Poor	0.374
Question_2_B_AI	Biostatistics	AI	0.808	-0.032	Too easy	Review	0.392
Question_4_B_AI	Cardiovascular - Heart failure	AI	0.808	0.018	Too easy	Poor	0.383
Question_10_B_AI	Female reproductive: Emergency Contraception	AI	0.808	0.132	Too easy	Fair	0.361
Question_20_B_AI	Cervical cancer screening	AI	0.740	-0.107	Appropriate	Review	0.409
Question_22_B_AI	Gastrointestinal - Abnormal liver function test	AI	0.726	0.388	Appropriate	Good	0.302
Question_28_B_AI	Immune mediated rheumatic diseases	AI	0.726	0.112	Appropriate	Fair	0.364
Question_8_A_AI	Sexually transmitted infections	AI	0.699	0.181	Appropriate	Fair	0.349
Question_24_A_AI	Cardiovascular - Cardiac murmurs	AI	0.699	-0.102	Appropriate	Review	0.410
Question_18_A_AI	Urology - UTI	AI	0.685	-0.124	Appropriate	Review	0.415
Question_30_B_AI	Renal - Nephrotic syndrome	AI	0.658	0.038	Appropriate	Poor	0.381
Question_27_A_AI	Respiratory - COPD	AI	0.630	0.227	Appropriate	Fair	0.337

Question_6_B_AI	Musculoskeletal - Rotator cuff	AI	0.562	0.217	Appropriate	Fair	0.338
Question_16_B_AI	Ortho, wrist or hand fracture	AI	0.562	0.153	Appropriate	Fair	0.354
Question_15_B_AI	Cardiovascular - Chronic Venous Insufficiency	AI	0.507	0.186	Appropriate	Fair	0.346
Question_10_A_AI	Dermatological - Eczema	AI	0.493	0.125	Appropriate	Fair	0.361
Question_12_B_AI	Emerging infections	AI	0.411	0.027	Appropriate	Poor	0.384
Question_26_A_AI	Neurological - Headache	AI	0.397	0.231	Appropriate	Fair	0.335
Question_21_A_AI	Preventive health - Smoking cessation	AI	0.370	0.143	Appropriate	Fair	0.357
Question_2_A_AI	Endocrine: Osteoporosis	AI	0.329	0.275	Appropriate	Fair	0.326
Question_22_A_AI	Ethics	AI	0.301	-0.307	Appropriate	Review	0.451
Question_23_B_AI	Hematological - Coagulopathy	AI	0.288	0.016	Appropriate	Poor	0.385
Question_13_A_AI	Psychiatric - Mood disorder	AI	0.164	0.239	Too difficult	Fair	0.342
Question_6_A_AI	Preventive health, travel medicine	AI	0.096	0.115	Too difficult	Fair	0.366
Question_14_B_Human	Psychiatric - Mood disorder	Human	0.986	0.073	Too easy	Poor	0.597
Question_7_B_Human	Preventive health, travel medicine	Human	0.973	0.333	Too easy	Good	0.585
Question_5_A_Human	Hematological - Coagulopathy	Human	0.932	0.208	Too easy	Fair	0.588
Question_25_B_Human	Dermatological - Eczema	Human	0.932	0.061	Too easy	Poor	0.598
Question_19_A_Human	Immune mediated rheumatic diseases	Human	0.890	0.385	Too easy	Good	0.572
Question_12_A_Human	Biostatistics	Human	0.863	0.258	Too easy	Fair	0.581
Question_29_A_Human	Diabetes Mellitus – Poorly controlled, Diabetic ketoacidosis	Human	0.863	0.196	Too easy	Fair	0.587
Question_24_B_Human	Ethics	Human	0.849	0.193	Too easy	Fair	0.587
Question_9_A_Human	Cardiovascular - Heart failure	Human	0.836	0.249	Too easy	Fair	0.581
Question_11_A_Human	Cardiovascular - Chronic Venous Insufficiency	Human	0.822	0.291	Too easy	Fair	0.577
Question_19_B_Human	Neonatal jaundice	Human	0.822	-0.036	Too easy	Review	0.609

Question_3_B_Human	Endocrine: Neck Lump	Human	0.781	-0.182	Too easy	Review	0.626
Question_7_A_Human	Gastrointestinal - Abnormal liver function test	Human	0.767	0.541	Too easy	Good	0.545
Question_17_B_Human	Preventive health - Smoking cessation	Human	0.767	0.213	Too easy	Fair	0.584
Question_26_B_Human	Respiratory - COPD	Human	0.753	0.097	Too easy	Poor	0.597
Question_28_A_Human	Renal - Nephrotic syndrome	Human	0.740	0.286	Appropriate	Fair	0.575
Question_4_A_Human	Ortho, wrist or hand fracture	Human	0.726	0.278	Appropriate	Fair	0.576
Question_17_A_Human	Neurological - Peripheral neuropathy	Human	0.726	0.077	Appropriate	Poor	0.600
Question_1_B_Human	Dermatological - Scabies	Human	0.726	0.348	Appropriate	Good	0.568
Question_9_B_Human	Neurological - Headache	Human	0.726	-0.005	Appropriate	Review	0.609
Question_15_A_Human	Female reproductive: Emergency Contraception	Human	0.699	0.142	Appropriate	Fair	0.592
Question_3_A_Human	Emerging infections	Human	0.685	0.258	Appropriate	Fair	0.578
Question_13_B_Human	Endocrine: Osteoporosis	Human	0.671	0.244	Appropriate	Fair	0.580
Question_21_B_Human	Diabetes Mellitus – Screening, Newly diagnosed	Human	0.589	0.077	Appropriate	Poor	0.601
Question_27_B_Human	Cardiovascular - Cardiac murmurs	Human	0.479	0.249	Appropriate	Fair	0.579
Question_29_B_Human	Urology - UTI	Human	0.479	0.003	Appropriate	Poor	0.611
Question_25_A_Human	Cervical cancer screening	Human	0.356	0.032	Appropriate	Poor	0.606
Question_5_B_Human	Sexually transmitted infections	Human	0.329	0.238	Appropriate	Fair	0.581
Question_11_B_Human	Gastrointestinal - Dyspepsia	Human	0.192	0.087	Too difficult	Poor	0.598
Question_1_A_Human	Musculoskeletal - Rotator cuff	Human	0.137	0.100	Too difficult	Fair	0.596

Annex S1. GenAI System Prompt for MCQ creation, utilizing ChatGPT 5 Thinking

You are an expert medical educator tasked with creating an extremely challenging and also inspired multiple-choice question for Family medicine specialists in training. Your goal is to test second-order thinking, emphasizing the application, analysis, and evaluation of knowledge based on Bloom's taxonomy about {topic}.

Please follow these steps to create the question, and utilise the necessary amount of tokens to do a good job:

- Take some effort to consider 15 testing points based on the {topic}, then choose 5 out of the 15, and generate 5 full MCQS.
- For every MCQ, give good distractors / choices, and a relevant clinical vignette with sufficient detail. Avoid pure recall questions. Vignette and choices should NOT reference inpatient settings (unless referring to emergency department)

1) Clinical Vignette

- Write a 120-word clinical scenario in the present tense.
- The scenario should be based in Singapore primary care
- Do NOT give patient names or clinic names or location names in the question
- Include only relevant details such as in presenting complaint, history, past medical history, drug history, social history, physical examination findings, bedside parameters, and necessary investigations to create a challenging MCQ
- History should have a narrative flavour, which physical examination can be objective.
- Use standard international units with reference ranges for any test results.
- STRICTLY do not hint/mention or reveal the diagnosis or include investigations that give away the answer.
- Ensure the clinical scenario tests higher-level skills of Bloom's taxonomy and second-order thinking.

2) Question

- Create a question that tests at least second order thinking skills.
- For example, formulate a question requiring the learner to first determine a diagnosis but then choose the right investigation or management plan.
- Keep the question stem concise and short.
- DO NOT summarize the clinical scenario or hint at any diagnosis.
- AVOID phrases like "In this patient with..." that telegraph the diagnosis.
- Do not ask for a wrong option out of possible correct answers

3) Multiple Choice Options

- Provide 5 options sorted in alphabetical order by the first word of each option.
- Include:
    - One best and correct answer

  - One correct answer, but not the best option
  - Three plausible options that might be correct, but are not the best answer
- Keep the length of all options consistent.
- Avoid options with more than 6 words
- Avoid non-functioning distractors
- Avoid misleading or ambiguously worded distractors.
- Avoid double barrelled answers.
- Ensure the correct answer has an equal chance of being option A, B, C, D or E.
- STRICTLY do not hint/mention/reveal the diagnosis or include investigations that give away the answer.

4) Correct Answer and Feedback:

- Identify the correct answer and explain why it is the best option.
- Provide option-specific explanations for why each option is correct or incorrect with clear reasoning.

Annex S2. MCQ-writing guidance and editing checklist

1) Key instructions

This document outlines the key instructions relating to the submission of the AKT-style MCQs. The AKT (Applied Knowledge Test) is the written examinations of both the GDFM (Graduate Diploma in Family Medicine) and the MMED(FM) (Master of Medicine in Family Medicine).

Each MCQ must contain a vignette relevant to family medicine setting (i.e. specifically in a family medicine setting and not in other settings). The vignette must be designed to test a particular key topic relevant to family medicine (i.e. avoid topics which are irrelevant to FM). There should be 5 options with only 1 correct answer. As far as possible the MCQ should be designed to test application of knowledge. As far as possible the difficulty of the MCQ should be pegged as close as possible to the level of the actual AKT. Questions of varying difficulty are encouraged but one should avoid deviating too far from the actual AKT standards (i.e. extremely easy or extremely difficult questions). A sample of 50 AKT MCQs are available in the shared drive for your reference. You are strongly encouraged to read the sample AKT to gauge the difficulty level required.

Below is an example of how a MCQ question should look like:

Figure S1: Example of a MCQ question

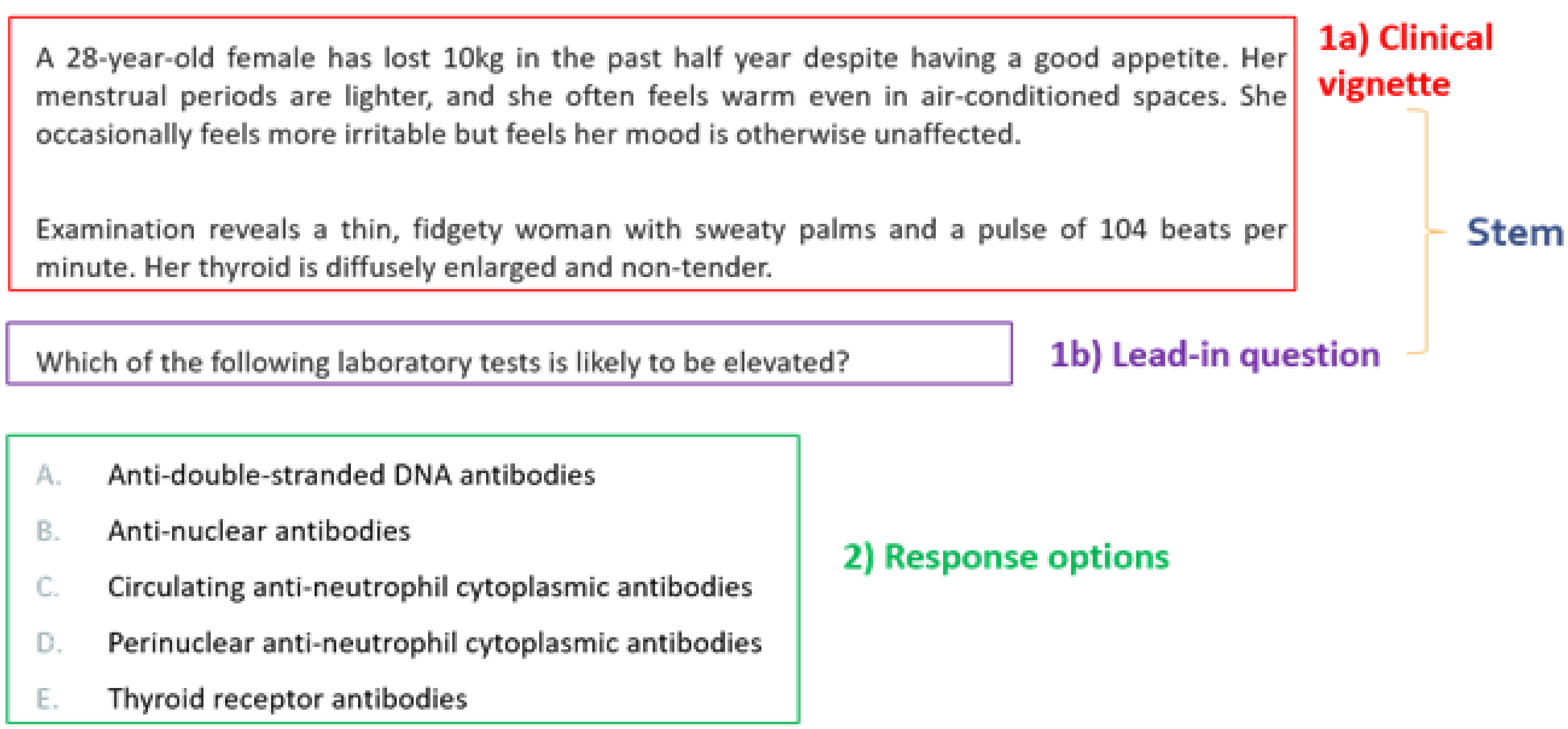

The MCQs may *feature additional information in the form of any of the following* [note the following list is *non-exhaustive and any form of images or clips relevant to the MCQ is acceptable*]:

- ECG image
- Dermatological image
- Spirometry image
- Table with laboratory values / investigations [with reference ranges if indicated]

- Retinal or eye image
- Otoscopy image
- X-ray and other radiological images
- Audio clips [such as cardiac murmurs, heart sounds, respiratory sounds on auscultations etc]
- Video clips [such as movement disorders, nystagmus, neurological signs etc]

2) Tips on Writing a Good MCQ

A) Coming up with a testing point

- It is recommended that you start by coming up with a testing point based on the assigned topic. For instance, if you are assigned the topic of Type 2 Diabetes, you may wish to consider whether to set the testing point as "diagnosis" or "management" [i.e. you wish to test if the candidate is able to apply the knowledge diagnostic criteria or apply knowledge on insulin initiation].
- For conditions which are common in Family Medicine such as Diabetes, the difficulty level of the question should be pegged at a higher level [such as a deeper dive into management]. For conditions which are uncommon in Family Medicine such as Rheumatoid Arthritis, the difficulty level of the question should be pegged at a lower level [such as recognition of the condition, rather than in-depth knowledge of therapeutics].

B) Writing the stem

- The stem is usually composed first and is best written as a complete sentence highlighting the reason for encounter. For example, it is preferrable to start the MCQ with "A 65 -year-old male presents for a review of his results done as part of the Screen for Life."
- The stem has to be relevant to clinical practice. Avoid using recall, hypothetical vignettes. Make it realistic and clinically relevant.
- Direct questions are preferred. For example, "Which of the following is the most likely diagnosis?" are clearer than sentence completions such as "The most likely diagnosis is …."
- A good stem is one in which the candidate must read the entire vignette in order to answer the question. Avoid writing questions which can be answered without requiring the candidate to read the vignette. For example, "Which of the following characteristics is a feature of benign pulmonary nodules?" can be answered without requiring the candidate to read the vignette.
- Other features of a good stem include the following:
    - Provide a complete statement
    - Include only relevant information

  - Have a length as short as possible, commensurate with the level of learning being tested [bear in mind that the test time for each MCQ is 90 seconds and examiners should avoid writing very long vignettes]
  - Ask for the correct, not the “wrong” answer [i.e avoid asking questions like “Which of the following is false?”, “All of the following are correct except?”]
  - Avoid absolute terms, such as always, never, all or none
  - Avoid imprecise terms, such as seldom, rarely, occasionally, sometimes, few or many
  - Avoid cues, such as may, could or can
  - Define eponyms, acronyms, or abbreviations when used
  - Avoid brand names such as “Augmentin” [use Amoxicillin-Clavulanate Acid]
  - Should pass the cover test – the stem should be answerable without all the options being read
  - Should test the application of medical knowledge rather than recall

<u>C) Writing the options</u>

- Each MCQ must be accompanied by 5 options.
- Features of good options include the following:
  - The options should be as short as possible. Avoid long options such as “Open airway, check breathing, commence CPR”
  - Follow grammatically from the stem / question
  - Relate to each other in kind (e.g. all diagnoses, all tests, all treatments).
  - Are similar in grammar, length and complexity, with distractors being plausible but clearly incorrect
  - Avoid “none of the above” and “all of the above”
  - Avoid imprecise terms such as frequently, sometimes and often
  - Follow a logical order (e.g. numeric, chronologic)
  - Are independent and do not overlap
  - Vary position of the correct answer (e.g. the correct answer should not always be option B)
  - Distractors should be plausible to a weak candidate – avoid non-functioning distractors; when testing conceptual understanding, distractors should represent common mistakes made by candidates.

D) Writing the explanation

- The explanation should be concise and to the point. Avoid writing long essays.
- The rationale for the correct response should be clearly explained. Similarly the rationale for the incorrect response should also be clearly explained.
- As far as possible, all explanations should come with the reference / source material on which the question is based and indicate this at the end of the explanation.

Figure S2. Total test scores for human-edited GenAI-assisted and educator-crafted postgraduate Family Medicine AKT-level MCQs in Singapore. Paired comparison of total scores among 73 complete-case participants. Each thin line connects one participant's GenAI-assisted and educator-crafted scores. The thick line connects group means; error bars represent 95% confidence intervals.

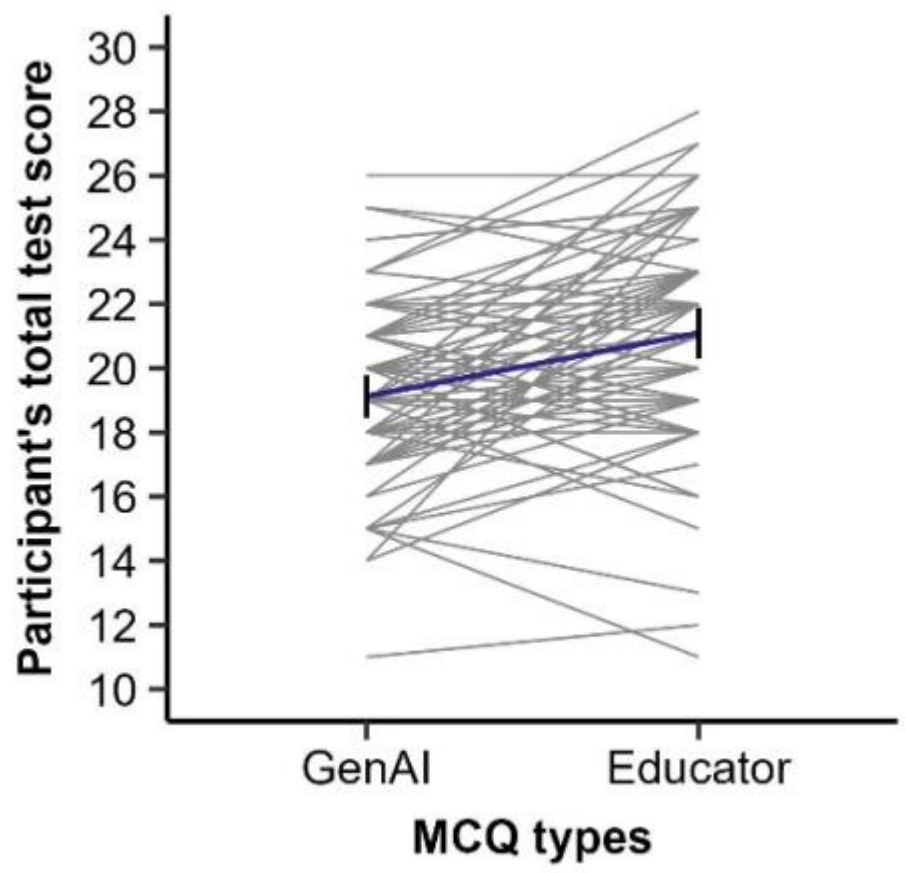

Figure S3. Alpha-if-item-deleted analysis for (A) GenAI-assisted and (B) educator-crafted MCQ items. Each bar represents the KR-20 value that would result from removing that item from the test. The dashed line indicates the overall KR-20 for the full item set.

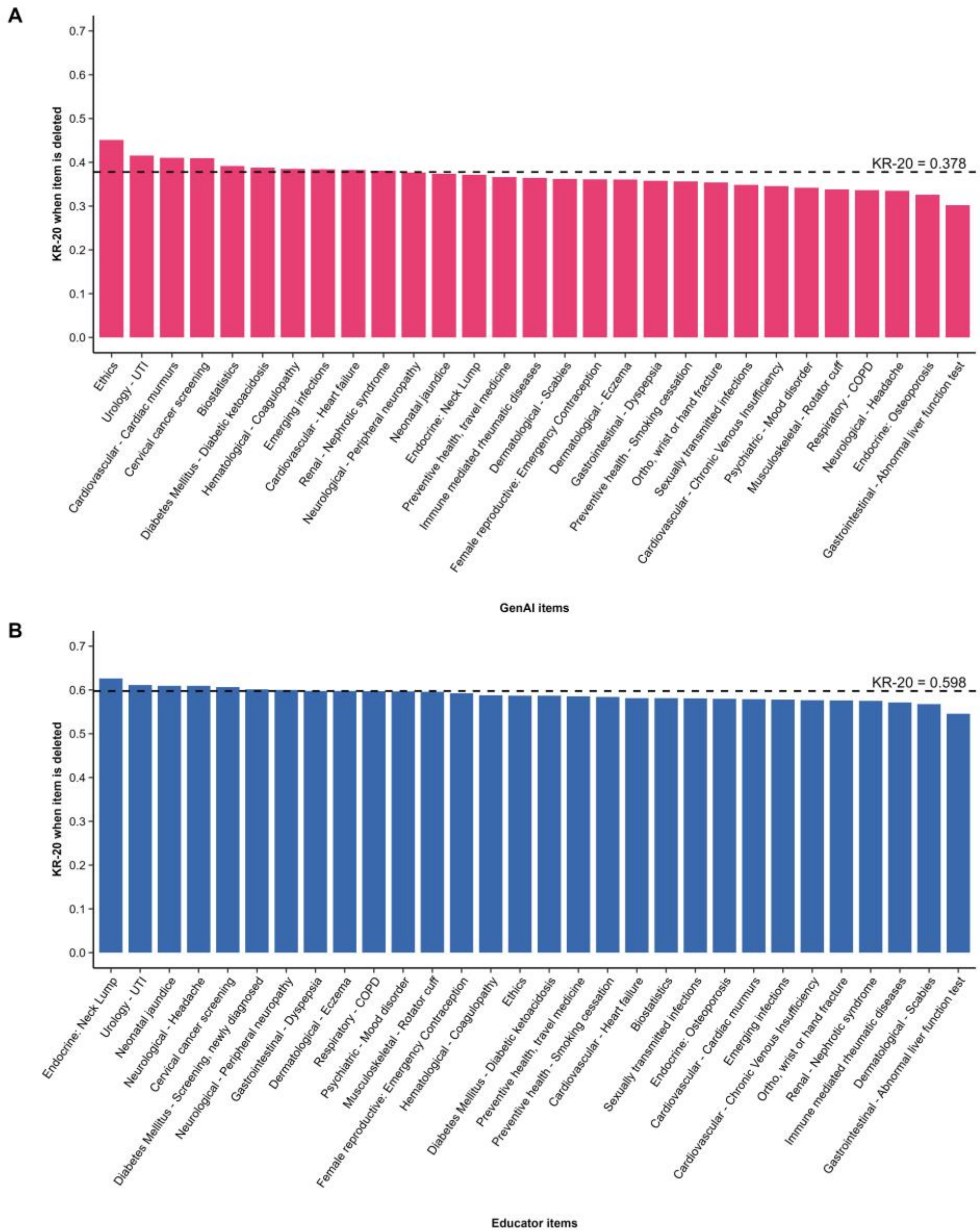

Figure S4. Relationship between participants' subjective difficulty ratings and objective item difficulty for GenAI-assisted and educator-crafted items.

Each point represents one item. Higher perceived difficulty ratings indicate items judged as harder, whereas higher difficulty index values indicate a greater proportion of correct responses. Negative correlations therefore indicate expected alignment between subjective and objective difficulty: items perceived as harder were answered correctly by fewer participants. GenAI-assisted items showed a strong negative correlation between perceived difficulty and objective difficulty (r=-0.72, *P*<.001), while educator-crafted items showed a moderate negative correlation (r=-0.54, *P*=.002).

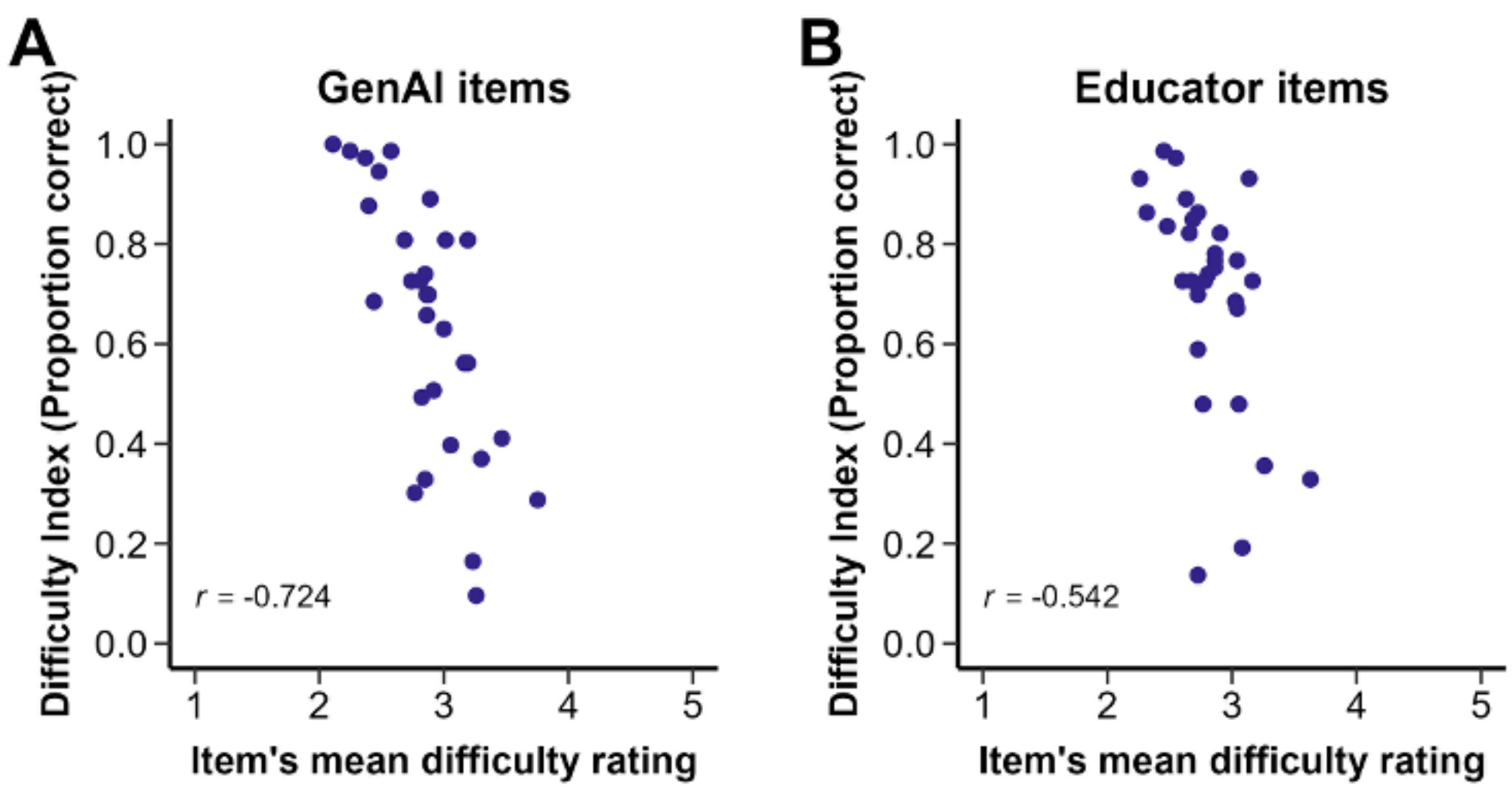

Supplement: Supplementary file 1 [file mededu-v12-e100179-s001.pdf]
